# Supplementary material for: Crystal Structure of Human CD47 in Complex with Engineered SIRPα.D1(N80A)
Source: Molecules. 2022 Aug 30;27(17):5574. doi: 10.3390/molecules27175574 (PMC9457805; doi:10.3390/molecules27175574)
Supplement: Supplementary file 1 [file molecules-27-05574-s001.zip › molecules-1841863-supplementary.pdf]

Supplemental file:

1) **Crystal mounting conditions**

**Crystal description**	**Condition/Cryo**	**Protein**	**Note**
IMM01-Fab/CD47his-CSHT-B5	well solution +20% glycerol	IMM01-Fab/CD47his	<4A
IMM01-Fab/CD47his-CSHT-B5	well solution +20% glycerol	IMM01-Fab/CD47his	<4A
IMM01-Fab/CD47his-PR-G4	well solution +20% glycerol	IMM01-Fab/CD47his	<4A
IMM01-Fab/CD47his-PR-G4	well solution +20% glycerol	IMM01-Fab/CD47his	<4A

2) **Diffraction test criteria.**

**Protein/Combination**	**Beamtime**	**note spot**	**Diffraction note**	**Observed resolution (Å)**	**Processed Space Group**	**Processed Resolution**
IMM01-Fab/CD47his-CSHT-B5	APS 19IDD	spots OK	single lattice	2.6	P212121	**2.76**
IMM01-Fab/CD47his-CSHT-B5	APS 19IDD	spots OK	few spots	3	P212121	3.23
IMM01-Fab/CD47his-PR-G4	APS 19IDD	spots OK	few spots	3.7	P422	3.94
IMM01-Fab/CD47his-PR-G4	APS 19IDD	spots OK	few spots	4.3	P422	6.39
